# Hands-on childcare garden intervention: A randomized controlled trial to assess effects on fruit and vegetable identification, liking, and consumption among children aged 3–5  years in North Carolina

**DOI:** 10.3389/fpsyg.2022.993637

**Published:** 2022-11-10

**Authors:** Nilda G. Cosco, Nancy M. Wells, Daowen Zhang, L. Suzanne Goodell, Muntazar Monsur, Tong Xu, Robin C. Moore

**Affiliations:** ^1^Department of Landscape Architecture and Environmental Planning, College of Design, North Carolina State University, Raleigh, NC, United States; ^2^Department of Human Centered Design, College of Human Ecology, Cornell University, Ithaca, NY, United States; ^3^Department of Statistics, College of Sciences, North Carolina State University, Raleigh, NC, United States; ^4^Department of Food, Bioprocessing and Nutrition Sciences, College of Agriculture and Life Sciences, North Carolina State University, Raleigh, NC, United States; ^5^Department of Landscape Architecture, Davis College of Agricultural Sciences and Natural Resources, Texas Tech University, Lubbock, TX, United States

**Keywords:** childcare, gardening, garden intervention, randomized controlled trial, healthy eating, diet, preschool, children

## Abstract

Gardening at childcare centers may have a potent influence on young children’s learning about fruits and vegetables and their development of healthy dietary behaviors. This randomized controlled trial examined the effect of a garden intervention on fruit and vegetable (FV) identification, FV liking, and FV consumption among 3–5-year-old children enrolled in childcare centers in Wake County, North Carolina, USA. Eligible childcare centers (serving primarily low-income families) were randomly selected and then randomly assigned to one of three groups: (1) intervention; (2) waitlist-control that served as a control in year 1 and received the intervention in year 2; or (3) no-intervention control. From the 15 participating childcare centers, 285 children aged 3–5  years were consented by their parents or guardians to participate. The intervention comprised six standardized, raised, mulched garden beds, planted with warm-season annual vegetables and fruits, and perennial fruits. A Gardening Activity Guide describing 12 age-appropriate, sequential gardening activities was distributed for teachers to lead hands-on gardening activities during the growing season. Data were gathered between Spring 2018 and Fall 2019. FV identification and liking were measured using an age-appropriate tablet-enabled protocol. FV consumption was measured by weighing each child’s fruit and vegetable snack tray before and after tasting sessions. Compared to children receiving no-intervention, children who received the garden intervention showed a greater increase in accurate identification of both fruits and vegetables as well as consumption of both fruit and vegetables during the tasting sessions. Consistent with prior research, the effects on fruit consumption were greater than on vegetable consumption. There was no significant effect of the garden intervention on children’s FV liking. Garden interventions implemented early in life foster learning about FV and promote healthy eating. Early exposure to gardening may yield a return on investment throughout the lifecourse, impacting healthy diet and associated health outcomes, which are particularly important within disadvantaged communities where children’s health is challenged by a host of risk factors. Clinical Trials Registration #NCT04864574 (clinicaltrials.gov).

## Introduction

Establishing fruit and vegetable (FV) consumption habits early in life may set children on a trajectory toward healthy eating, helping them to maintain healthy weight and reduce the later risk of obesity and associated health issues (Birch et al., 2007; Schwartz et al., 2011; Grimm et al., 2014). Experiential learning in early childhood is central to child development and, therefore, may be a critical strategy to engage young children in increasing about fruit and vegetables (FV) by tasting and exploring through hands-on activities (Nekitsing et al., 2018; Varman et al., 2021).

Contact with fresh produce is important to enable cognitive and other developmental processes that may help to build a sensory repertoire of food attributes (i.e., textures, flavors, smells, colors, shapes) while individual food preferences evolve (Zeinstra et al., 2007). Children’s progressive knowledge of FV may be extended through hands-on experiences across a range of gardening activities: planting, caring, harvesting, preparing, and eating (Parmer et al., 2009). Gardening may be the most effective way for children to participate in food production (Cooke, 2007) which, in turn, has been linked to healthy dietary intake (Savoie-Roskos et al., 2017; Skelton et al., 2019).

Children who grow their own FV are more likely to eat garden produce (Cabalda et al., 2011; Namenek Brouwer and Benjamin Neelon, 2013). Moreover, Langellotto and Gupta (2012) suggest that garden-based learning may have a greater impact on fruit and vegetable consumption than nutrition education programs alone. There are various mediating mechanisms or pathways through which garden-based experiential learning might plausibly affect children’s intake of FV. These pathways include accessibility of FV (Cullen et al., 2003); daily exposure (Cooke, 2007); familiarity with local FV (Bevan et al., 2016; Nekitsing et al., 2018); and availability of FV (Jago et al., 2007).

Timing of garden interventions is critical because early introduction of FV may support retention of habitual FV intake (Birch et al., 2007). Review of potential predictors of children’s FV consumption (Cooke, 2007) shows age of introduction inversely correlated with FV intake in preschool-age children (Cooke et al., 2004). Early introduction of FV may also minimize food neophobia (i.e., dislike or nonacceptance of new food) in preschool years (Cooke et al., 2004) and the introduction of non-taste sensory learning about fresh produce (e.g., planting, harvesting, etc.) may support familiarity with fruit and vegetables not offered at home (Nekitsing et al., 2018).

In this study, gardening conducted as an early childhood experiential process is considered a potential conduit for establishing healthy food preferences that support FV intake. While prior research suggests that gardening may affect school-age children’s learning (Berezowitz et al., 2015; Wells et al., 2015) and diet (Davis et al., 2015; Skelton et al., 2019), few studies have focused on the influence of garden interventions on preschool-age children, when effects may be particularly potent. Moreover, many prior studies face methodological limitations such as short duration, small sample sizes, absence of a control group, or lack of random assignment, which compromise causal conclusions (i.e., internal validity; Ohly et al., 2016; Savoie-Roskos et al., 2017; Landry et al., 2021). The goal of this randomized controlled trial (RCT) is to increase understanding of the impact of hands-on gardening on preschool children’s FV knowledge, FV liking, and consumption of FV during snack sessions.

This study examines three key research questions among children aged 3–5 years enrolled in childcare centers: (1) Does the garden intervention affect children’s FV identification? (2) Does the garden intervention affect FV preference (“liking”)? (3) Does the garden intervention affect FV consumption during tasting events?

## Materials and methods

### Research design

This randomized controlled trial employed a waitlist-control design to assess the impact of the Preventing Obesity by Design (POD; Moore and Cosco, 2014) garden intervention on FV identification, FV liking, and FV consumption among children aged 3–5 years, enrolled in 15 childcare centers in Wake County, North Carolina.

The research design is illustrated in Table 1. Fifteen childcare centers were randomly assigned to one of the following groups: Group 1 intervention (5 centers, ~100 children), to receive the garden intervention in Year 1; Group 2 waitlist control or “delayed intervention” (5 centers, ~100 children), to participate as control group in Year 1 and to receive the garden intervention in Year 2; or Group 3, no-intervention control (5 centers, ~100 children) that joined the study in Year 2 and received the garden installation and training resources after completion of data collection.

**Table 1:** Research design with intervention, waitlist (delayed intervention), and control groups.

	Year 1				Year 2			
Random assignment:	Winter	Spring	Summer	Fall	Winter	Spring	Summer	Fall
Group 1: intervention (5 sites, 100 children)		O_1_	X	O_2_				
Group 2: waitlist (5 sites, 100 children)		O_1_		O_2_		O_3_	X	O_4_
Group 3: control (5 sites, 100 children)						O_1_		O_2_

O, observation (data collection). X, garden intervention.

Data collection occurred in the Spring of Year 1 for Groups 1 and 2. The initial intervention centers (Group 1) received the garden intervention in the summer of Year 1 and both Groups 1 and 2 participated in data collection again in the early Fall, following the intervention. In Year 2, data were collected from Groups 2 and 3 in the Spring. Group 2, comprising the five waitlist control centers, then received the garden intervention in the summer of Year 2. Data were then collected from Groups 2 and 3 in early Fall of Year 2. The trial proceeded without deviation from its design: no modifications or outcome changes were made after the trial commenced and the trial was not stopped or ended prematurely. Protocol details, recruitment strategy, and participant characteristics of this RCT are reported elsewhere (Cosco et al., 2021). The study is registered with ClinicalTrials.gov, #NCT04864574. The research design and methods were approved by the North Carolina State University Institutional Review Board (IRB), protocol approval #5908.

### Childcare center recruitment

Study sites were identified in collaboration with the Wake County Smart Start, NC, from a pool of approximately 310 licensed childcare centers within the county. Based on the eligibility criteria, presented in Table 2, Wake County Smart Start invited 23 centers to complete an online application that included verification of eligibility criteria, demographic characteristics of the center, and a statement indicating willingness to work collaboratively with the research team. Of the 23 invited centers, 15 were deemed to meet the requisite criteria. The 15 centers were randomly assigned to Groups 1, 2, and 3, as described above. The study team met with childcare center directors and preschool teachers to review the project aims and expectations and to verify willingness to collaborate. Directors agreed to include their centers in the study by signing a letter that described the garden intervention.

**Table 2:** Childcare center eligibility criteria.

(1) Assigned a 4 or 5 Star Rated License by NC Division of Child Development & Early Education (DCDEE)
(2) Serve a majority of children eligible for the Wake County Childcare Subsidy Program
(3) Contain at least two preschool classrooms (3-5-year-old children)
(4) Enrollment size within the middle third for Wake Co (excluding smallest and largest centers)
(5) Operate a regulated on-site kitchen to prepare food for snacks
(6) Employ cooking staff
(7) Operate a year-round calendar
(8) Own or lease current space for at least 5 years into the future
(9) Do not currently conduct on-site FV gardening but interested in implementing in the future

Of the 15 selected centers, nine facilities were owned by the organization, and six were leased. Most centers were well established at their sites showing a tenure range between 5 years and permanent location (10 of the 15 centers declared operating at the current site for 10 years or more). The category of operation was declared as “independent” (10 centers) or “franchise” (5 centers). The average area of center outdoor spaces (8,458 sq. ft.) reflected North Carolina licensing requirements for enrollment size of each selected site. The menus at all childcare centers adhered to the North Carolina Child Care Rules, General Nutrition Standards rule 10A NCAC.0900 (State of North Carolina Office of Administrative Hearings, 2012).

Enrollment data for North Carolina’s regulated childcare centers at the time of recruitment (North Carolina Department of Health and Human Services, 2017) indicated an average enrollment of 70 children, 15% of whom received subsidies. Selected study childcare centers had an average enrollment of 63 children, of whom 51% received subsidies (more than three times the state average to match study goals).

A total of 543, 3–5 years old children were eligible from the pool of 15 selected childcare centers. Of those, 285 children were consented by parents to participate in the study. The sample size was determined by a power analysis calculation as described in Cosco et al. (2021). At baseline, mean age of children was 3.26 years (SD = 0.57), BMI was 16.12 (SD = 1.46), and 64.7% were non-white. In Year 2, additional children were recruited to account for the loss of graduating children and unstable enrollment. Attrition of children from the study occurred at a rate of 23% per year. Children who had incomplete data were included in the analyses (as is the convention and advantage of general linear mixed models). Thus, the overall number of children included in analyses was 285.

### Participants: Children and RCT groups

At baseline, the sample comprised 250 children, mean age 3.26 years (SD = 0.57); 48.8% male; 64.7% non-white; and mean BMI 16.12 (SD = 1.46). Characteristics of the three randomized groups are summarized in Table 3. Group 1 (Year 1 intervention) comprised 61 children, mean age 3.17 years; 50.80% male; 58.90% non-white; 44.30% receiving subsidies; and mean BMI 16.13. Group 2, the Waitlist control (Year 2 intervention) included 119 children, mean age 3.15 years; 44.90% male; 62.20% non-white; 47.90% receiving subsidies; and mean BMI 16.20. Group 3, the Control group comprised 70 children, mean age 3.51 years, 53.60% male, 71.90% non-white, 62.30% receiving subsidies, and mean BMI 15.97. Non-white children include African American, Asian, Latino, and Multi-racial. Participating children were at healthy weight showing similar BMI means by group.

**Table 3:** COLEAFS randomized group characteristics at baseline, by intervention (I), waitlist (delayed intervention) (W), and control centers (C).

Group	*n*	Age *xˉ* (sd)	% Male	% Non-white	% Subsidy	BMI *xˉ* (sd)
1. Interv	61	3.17 (0.53)	50.80%	58.90%	44.30%	16.13 (1.31)
2. Waitlist	119	3.15 (0.55)	44.90%	62.20%	47.90%	16.20 (1.63)
3. Control	70	3.51 (0.56)	53.60%	71.90%	62.30%	15.97 (1.27)

### Constructs and measures

Below, the operationalization of the study’s independent and dependent variables is described. Additional details can be found in Cosco et al. (2021).

#### Independent variable: The garden intervention

**Figure 1:**
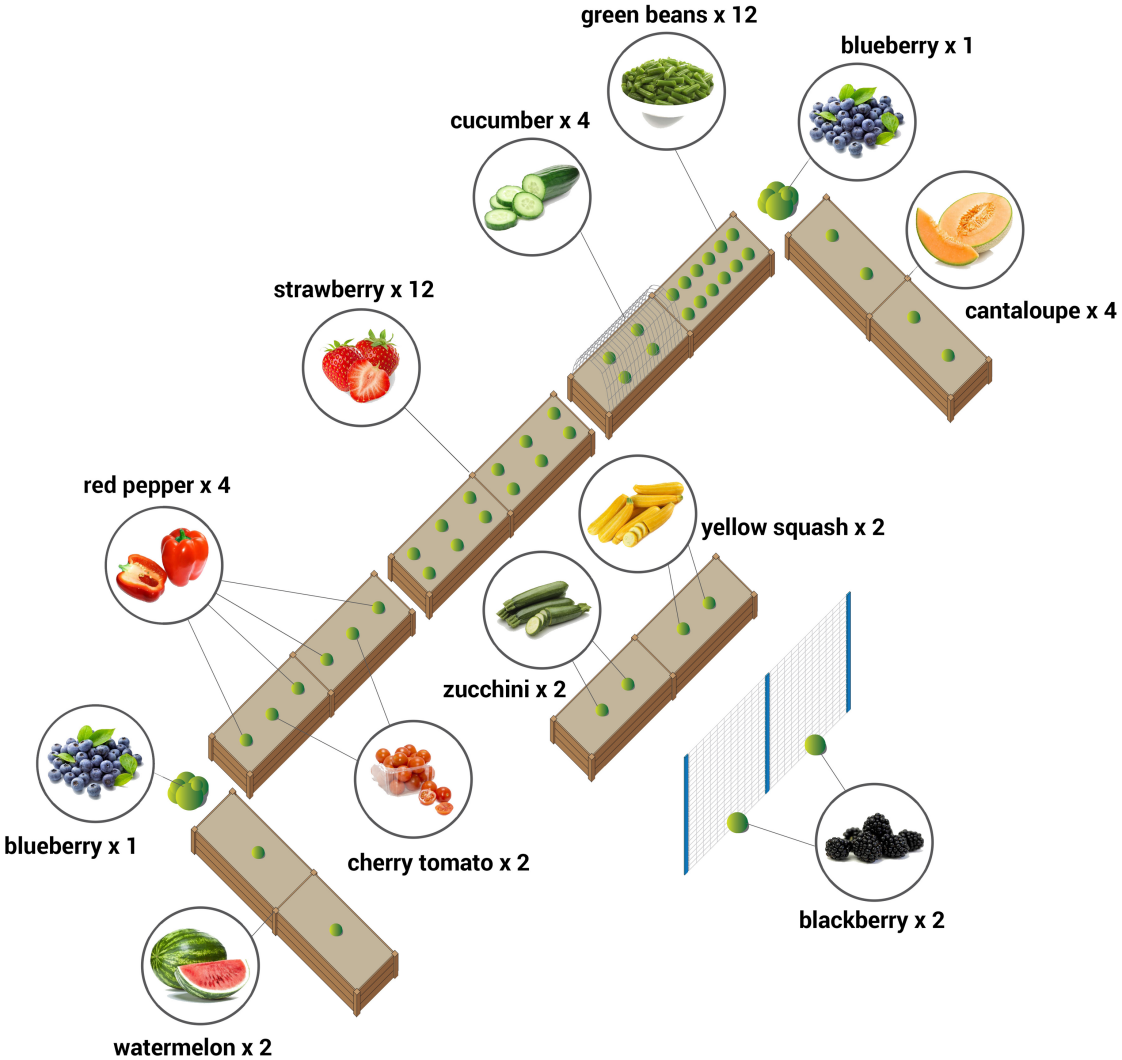
Standard garden layout (re-configured on-site to conform to spatial constraints as necessary).

The garden intervention (Preventing Obesity by Design (POD) Garden Component), comprised six raised beds, prescribed FV plantings (Figure 1), a seasonal planting regime, garden engagement activities, and weekly technical assistance. The six vegetables (cucumbers, green beans, green peppers, tomatoes, yellow squash, and zucchini) were selected because all have a long harvest season extending into August in the Piedmont region of North Carolina. Five fruits (blackberries, blueberries, cantaloupe, strawberries, and watermelon) were selected. Blueberries (two shrub varieties) and blackberries (two vines on trellis) were planted in-ground. Because the strawberry harvest is early in the year and both blueberries and blackberries have modest yields in the first year after planting, the intervention was augmented with purchased berries for the snack sessions. Although apples were included in the tasting session, they were not included in the garden installation because tree fruits take too long to produce.

As described by Cosco et al. (2021), the intervention also included “The Garden Activity Guide” comprising 12 age-appropriate activities to be led by the teacher, who was instructed to use the Guide to plan their daily outdoor activities. There were four activities in each of three categories: Preparing, Caring, and Harvesting/Eating. The 12 activities ensured that children were regularly engaged with the garden from the preparatory phases of examining and sprouting seeds to harvesting, preparing, snacking, and taking home produce. Teachers delivered up to seven of the 12 activities per week (e.g., examining seeds, preparing beds, watering, weeding, and snacking). Typically, three to four activities occurred each week over a period of 13 weeks. Activities were usually carried out during outdoor time and lasted about 30 min. Childcare centers retained the activity booklets and installed gardens upon completion of the study.

#### Demographic variables

Several demographic variables were measured at the level of the individual child (i.e., age, gender, and BMI) and at the level of the childcare center (e.g., teacher education, parental education, and staff race/ethnicity).

#### Dependent variables

Each of the dependent variables described below is calculated for fruit (F), for vegetables (V), and for FV combined.

*Fruit & Vegetable Identification.* FV identification was measured by asking if the child knew (Yes/No) each of the 12 FV shown on a tablet screen (iPad). The child was then asked, verbally, to name the item and their response was recorded manually by the research assistant. This process yields three dependent variables: fruit identification, vegetable identification, and FV identification.

*Fruit & Vegetable Liking.* FV liking was measured using a digital version of the picture-based survey developed by Carraway-Stage et al. (2014). When presented with an image of a fruit or vegetable, the child is instructed “Tell me if you like or do not like this food by pointing to one of the faces” and responds by pointing at the 5-point emoticon scale presented on a tablet (iPad) where 5 = super yummy, 4 = yummy, 3 = just okay, 2 = yucky, and 1 = super yucky. The original Fruit, Vegetable Preference Measure (Carraway-Stage et al. 2014) has strong internal consistency (alpha = 0.79) and acceptable test–retest reliability (with 7–14 days between administrations) for the 9-item fruit scale (*r* = 0.51), the 10-item vegetable scale (*r* = 0.40) and the combined FV scale (*r* = 0.49). The measure used in the current study includes images of six fruits and six vegetables and yields three dependent variables: Fruit liking (sum of 1–5 ratings for 6 fruits), range 6–30; vegetable liking (sum of 1–5 ratings for 6 vegetables), range 6–30; and FV liking (sum of 1–5 ratings for all 12 FV), range 12–60.

**Figure 2:**
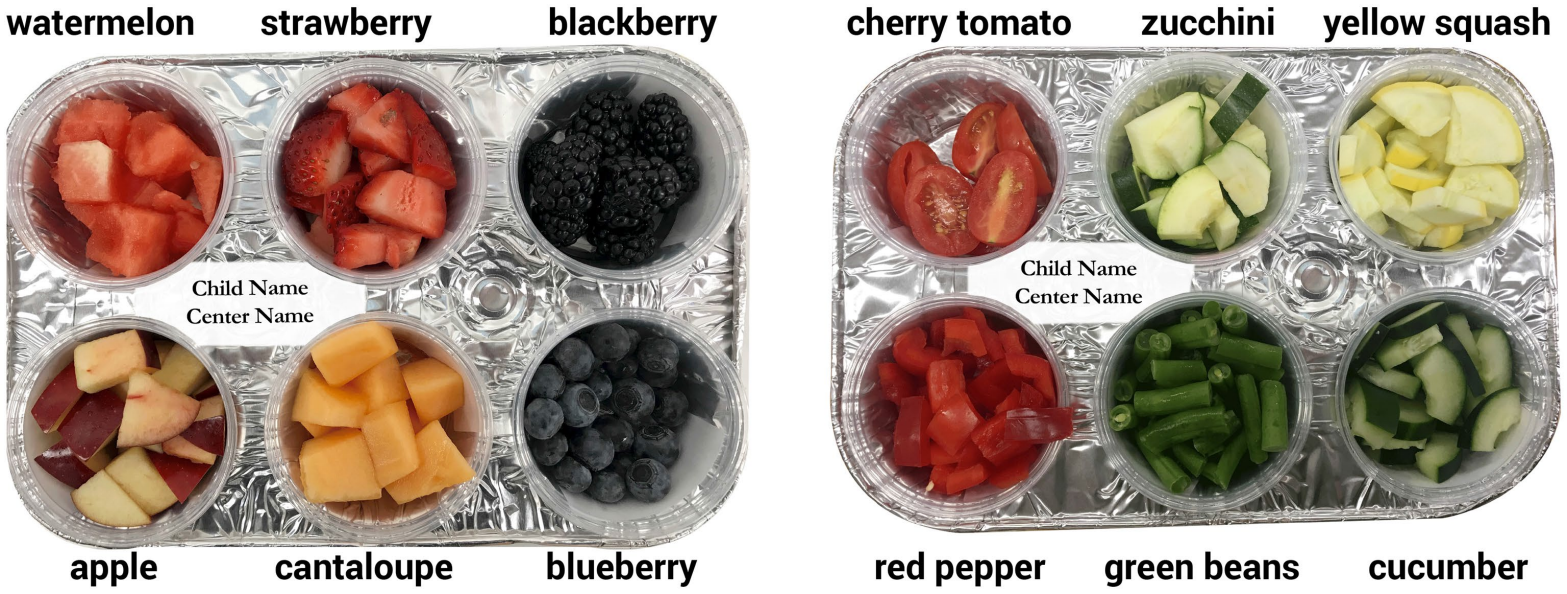
Prepared snack trays presenting approximately 300 grams of F or V to each child.

**Figure 3:**
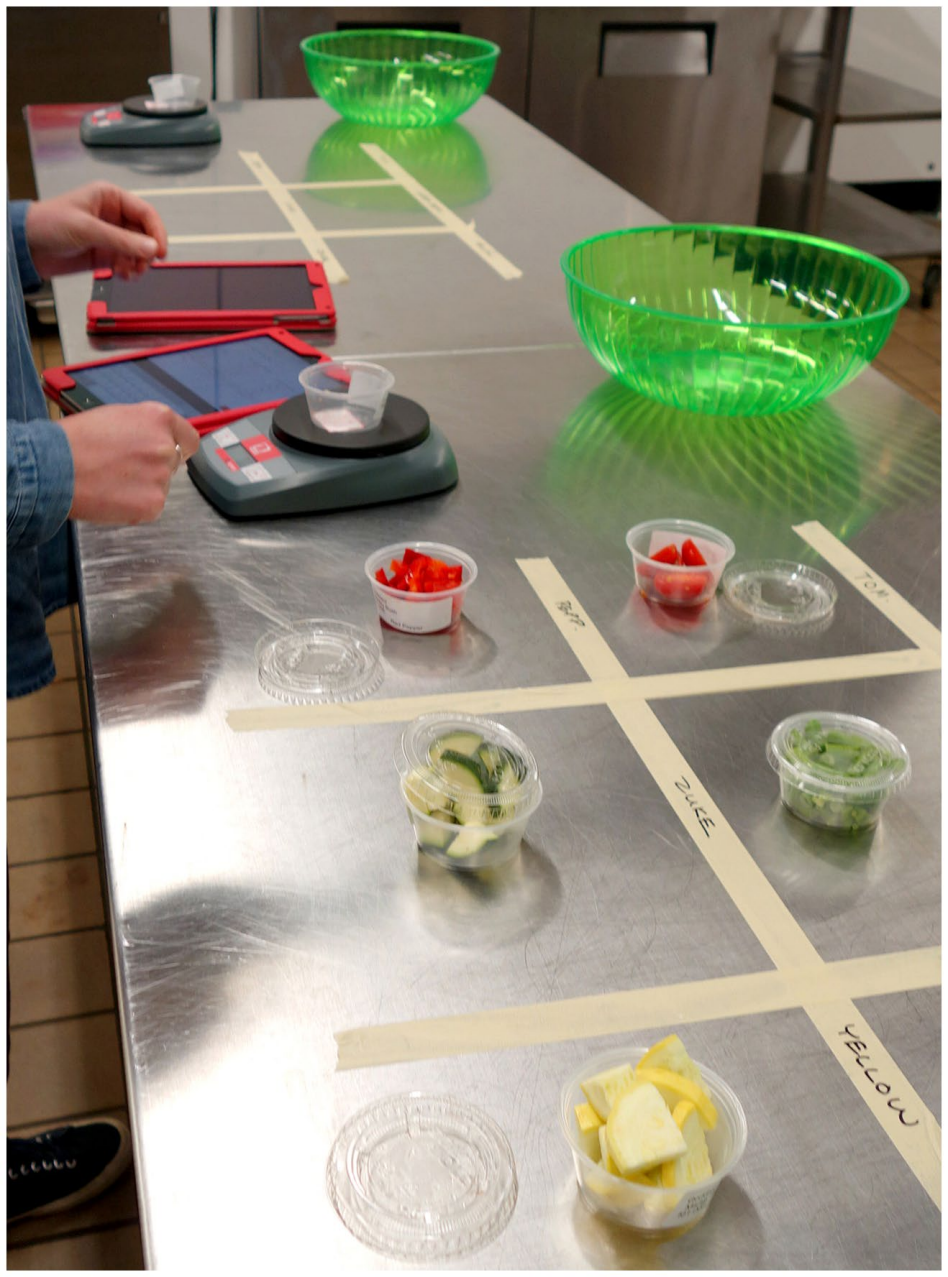
Tray preparation and weighing table.

*Fruit & Vegetable Consumption.* FV consumption was measured (see Cosco et al., 2021), using a protocol derived from that of Witt and Dunn (2012). While they presented children with 1 cup of mixed fruit or mixed vegetables and included a small container of ranch dressing on the day vegetables were eaten, in this study we presented children with 6 individual cups, each containing approximately 50 grams of each fruit or vegetable (without dressing), in two (6′ × 12′) 6-compartment trays (each approximately 300 grams) labeled with child’s name and ID number (Figure 2). The vegetable snack session was held 1 day prior to the fruit snack session. Each of the six fruit or six vegetable servings was weighed (grams) on a Tanita HD-357 scale before serving and after the snack period and data entered on the iPad (Figure 3). Uneaten food was composted. Each child’s consumption was calculated for fruit, for vegetable, and for FV combined by subtracting the weight remaining on the tray from that served. This yields three consumption measures: F in grams (of approximately 300 grams served), V in grams (of approximately 300 grams served), and FV in grams (of approximately 600 grams served).

The dependent variables were measured on different days within 1 week. On Monday, a storytelling session was held to familiarize the students with the data collection tablets and response options. On Tuesday, FV identification and FV liking data were gathered using the tablets. On Wednesday and Thursday, respectively, vegetable and fruit snack sessions were held, and V and F consumption data were collected.

### Analytic strategy

The three core research questions concern the effect of the childcare garden intervention on (1) FV identification; (2) FV liking; and (3) FV consumption. These questions are addressed by examining 9 key dependent variables, as described above (i.e., F, V, and FV identification; F, V, FV liking; F, V, and FV consumption). For each outcome variable, a linear mixed model approach is used with childcare center random effect and nested child random effect within childcare center to estimate the true mean score of each outcome for Spring (pre-intervention) and Fall (post-intervention) for each year (2018 and 2019; MIXED procedure of SAS software v 9.4, SAS Institute, Inc., Cary NC, United States). An intervention effect for an outcome in a particular year is defined as the difference of the pre-post true mean score changes between groups receiving intervention and those receiving no intervention (i.e., difference-in-difference). An approximate t-test for contrasts in linear mixed models was conducted to test the equality of two intervention effects for each outcome variable of interest in Year 1 and 2 and no statistically significant difference was found. Therefore, we assume equal intervention effects for outcomes and present the estimated intervention effects and their standard errors in Table 4.

**Table 4:** Estimated intervention effect (standard error) for each outcome variable from a hierarchical linear mixed effect model, with assumption that year 1 and year 2 effects are the same.

Outcome	Est. Effect (SE)	Value of *p*
*FV identification*		
F_I	0.40 (0.17)	0.022^*^
V_I	0.87 (0.27)	0.002^**^
FV_I	1.26 (0.39)	0.001^**^
*FV liking*		
F_L	0.14 (0.14)	0.304
V_L	0.07 (0.17)	0.667
FV_L	0.27 (0.25)	0.283
*FV consumption: grams (“CG”)*		
F_CG	24.99 (8.65)	0.004^**^
V_CG	14.08 (3.68)	<0.001^***^
FV_CG	37.87 (10.91)	0.001^**^

^*^p < 0.05, ^**^p < 0.005, ^***^p < 0.001.

## Results

### Examining the research questions

Table 5 presents the observed pre-post sample means scores and their change for each outcome in Year 1 and Year 2. Note that because of dropouts from enrolled children, the sample mean scores may not be unbiased estimates of the corresponding true mean scores; hence, the observed intervention effect may not be an unbiased estimate of the true intervention effect.

**Table 5:** Pre-post sample mean scores and their change for intervention and no-intervention groups in Year 1 and 2 for each outcome (*n* = 285).

Variables	Year 1 Intervention			Year 1 Control			Year 2 Intervention			Year 2 Control		
	Pre	Post	Change	Pre	Post	Change	Pre	Post	Change	Pre	Post	Change
FV Identification												
F_I	4.86	5.13	0.27	4.84	4.97	0.13	4.66	5.38	0.72	5.29	5.27	−0.02
V_I	3.88	5.13	1.25	3.54	4.15	0.61	3.79	4.96	1.17	4.51	4.55	0.04
FV_I	8.73	10.23	1.50	8.40	9.12	0.72	8.44	10.34	1.90	9.81	9.82	0.01
FV Liking												
F_L	2.47	2.72	0.25	2.56	2.50	−0.06	2.47	2.43	−0.04	2.62	2.60	−0.02
V_L	3.11	3.56	0.45	3.12	2.98	−0.14	3.11	2.96	−0.15	2.91	3.21	0.30
FV_L	5.62	6.27	0.65	5.82	5.50	−0.32	5.63	5.39	−0.24	5.53	5.82	0.29
FV Consumption (grams)												
F_CG	102.78	126.62	23.84	135.32	128.9	−6.43	120.20	128.67	8.47	145.94	116.41	−29.54
V_CG	12.44	22.2	9.76	28.80	26.35	−2.45	27.92	32.15	4.24	32.15	14.30	−17.85
FV_CG	112.23	142.94	30.71	163.48	153.28	−10.20	147.46	148.60	21.14	179.59	128.41	−51.18

F_I, Fruit Identification [0–6]; V_I, Vegetable Identification [0–6]; FV_I, Fruit + Vegetable Identification [0–12]. F_L, Fruit Liking [1–5]. V_L, Vegetable Liking [1–5]; FV_L, Fruit + Vegetable Liking [2–10]; F_CG, Fruit Consumption grams [0–300]; V_CG, Vegetable Consumption grams [0–300]; FV_CG, Fruit + Vegetable Consumption grams [0–600]. Descriptive statistics for illustrative purposes only. Because the center is the sampling unit, and the child is the unit of analysis, regular SE or CI are not reported. For variability of parameters of interest, see Table 4.

**Figure 4:**
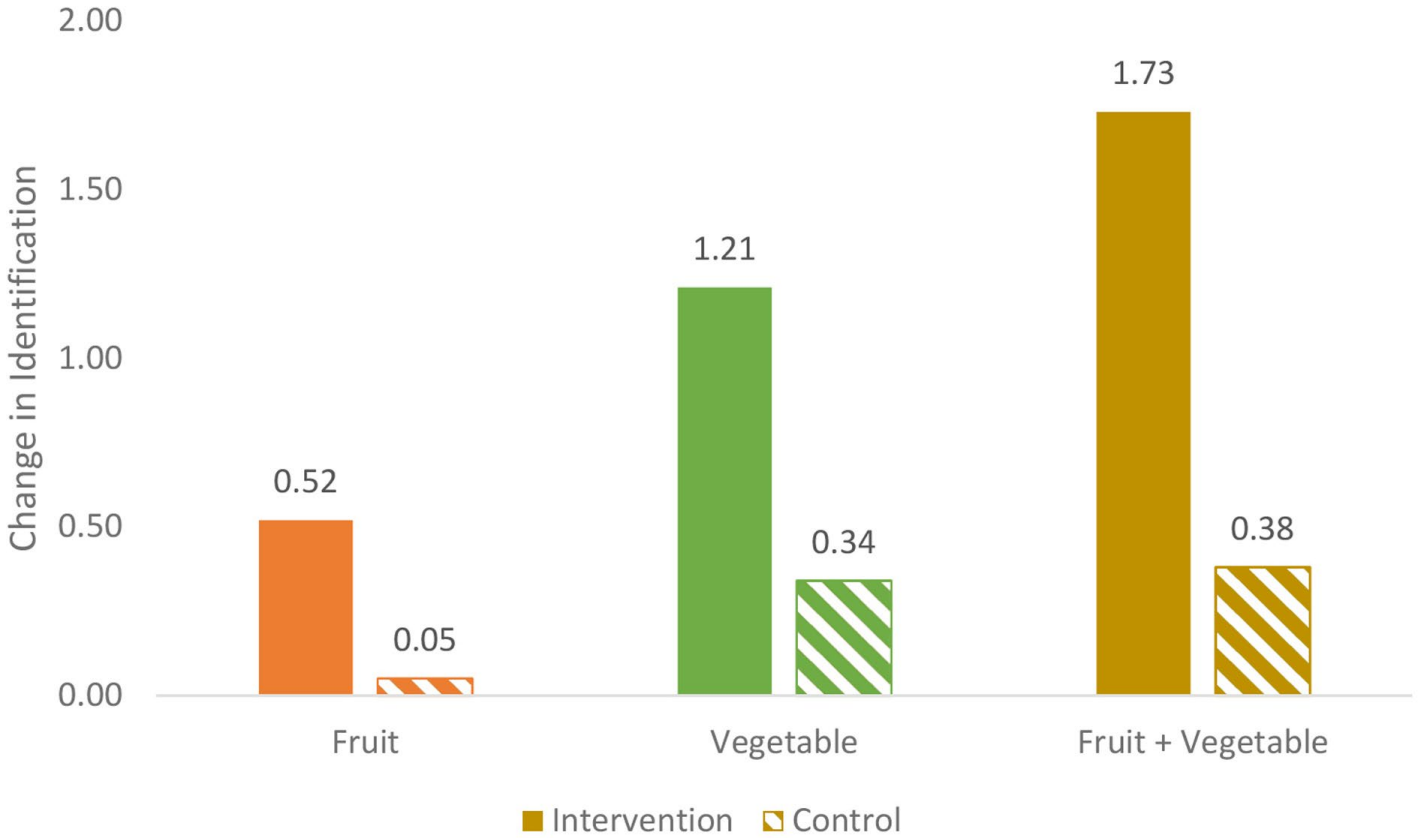
Pre-post change for intervention v. no intervention for F, V, and FV identification (number of FV).

**Figure 5:**
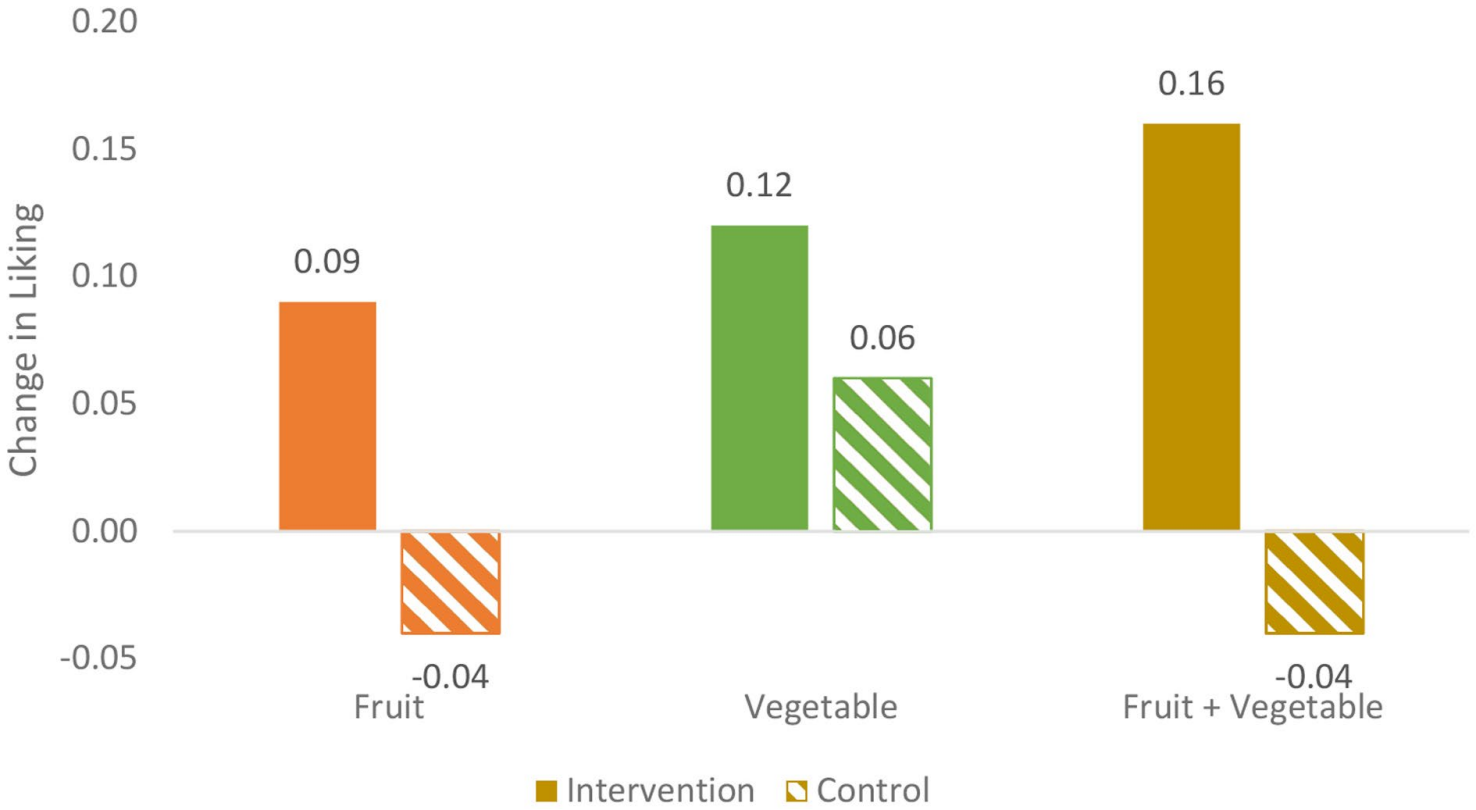
Pre-post change for intervention v. no intervention for F, V, and FV liking (ratings 1-5 of 6 F, 6 V).

**Figure 6:**
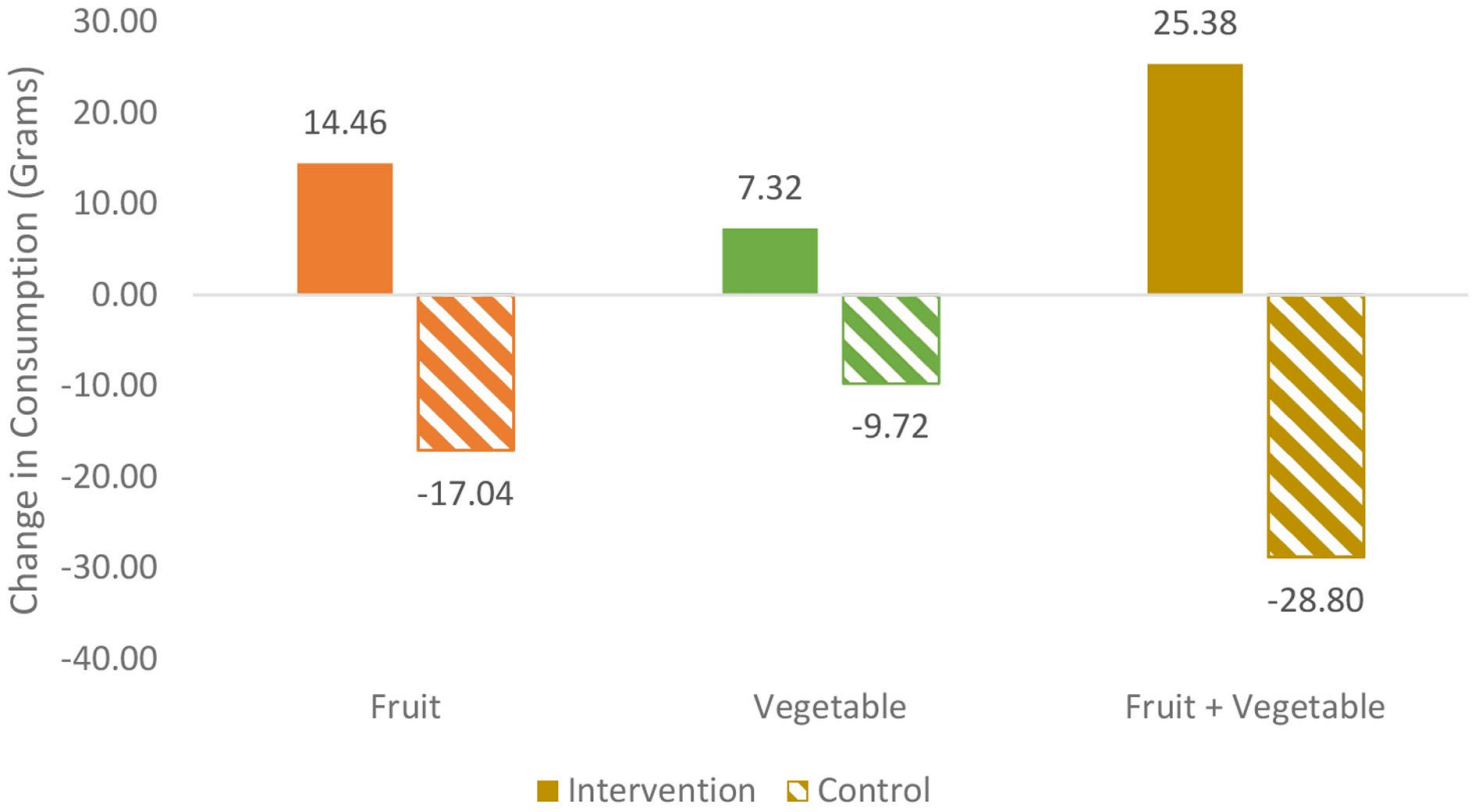
Pre-post change for intervention v. no intervention for F, V, and FV consumption in grams during snack sessions (of 300 g. F, 300 g. V served).

Below, each research question is addressed regarding FV identification, FV liking, and FV consumption. Figures 4–6 illustrate the intervention versus no-intervention observed pre-post change mean scores for FV identification, FV liking, and FV consumption, respectively. The estimated intervention effects for each outcome variable are presented in Table 4.

#### FV identification

*Does the garden intervention affect children’s FV identification?* As shown in Table 5 and Figure 4, both the intervention and the no-intervention groups show increases in V, F, and the combined FV identification from baseline to follow-up, the increases demonstrated by the intervention group are consistently greater than those of the control (no-intervention) group. Thus, difference-in-difference (i.e., changes in intervention data v. changes in no-intervention data) trends are in line with the hypotheses.

In fact, the estimated intervention effects are statistically significant for all three variables: F, V, and combined FV identification, as presented in Table 4. Compared to children receiving no intervention, children in the intervention group are expected to identify 0.4 more individual fruits, 0.87 more vegetables, and 1.26 more FV combined. This is equivalent to an increase of about half of a fruit and nearly one vegetable identification. These estimated effects are significant at the *p* < 0.05, *p* < 0.005 and *p* < 0.005 level, respectively.

#### FV liking

*Does the garden intervention affect FV liking?* As shown in Table 5 and Figure 5, changes in FV liking show a less consistent pattern than those for FV identification. In Year 1, difference-in-difference trends are in line with the hypotheses, i.e., the intervention group increases from pre- to post-intervention and the control group decreases in F, V, and FV liking. However, in Year 2, the opposite trend is apparent, when the intervention group results decrease from pre- to post-intervention and the no-intervention control group results increase (see Table 5). Thus, because the intervention effects differ from Year 1 to 2, the estimated common effect is not significant for F, V, or FV, as shown in Table 4; with *p*-values of 0.30, 0.67, and 0.28, respectively.

#### FV consumption

*Does the garden intervention affect FV consumption during a tasting event?* As shown in Table 5 and Figure 6, the intervention group consistently shows increases in F, V, and FV consumption while the no-intervention group shows decrease in the three variables from pre- to post-intervention.

The difference-in-difference is statistically significant for all three consumption measures. As shown in Table 4, compared to children receiving no intervention, children who received the garden intervention are expected to eat 25 grams more fruit and 14 grams more vegetables during snack time (and about 38 grams more FV combined). These estimated effects are significant at the *p* < 0.005, *p* < 0.001, and *p* < 0.005 level, respectively.

## Discussion

### Conclusion and interpretation

The childcare garden intervention had significant positive effects on children’s learning to identify both fruit and vegetables and on their consumption of fruit and vegetables during a tasting session. There was no significant effect on children’s liking of fruit or vegetables. These findings are largely consistent with prior research. Regarding FV identification, studies conducted primarily with elementary school students (ages 7–10 years), suggest that gardening can bolster children’s science learning including FV knowledge (Parmer et al., 2009; Berezowitz et al., 2015; Wells et al., 2015).

The present study extends the evidence to preschool children (ages 3–5 years). With respect to FV consumption, findings align with previous research that suggests hands-on gardening may modestly boost children’s FV consumption (Namenek Brouwer and Benjamin Neelon, 2013). Moreover, the current finding that the garden intervention had a stronger effect on fruit rather than on vegetable consumption is consistent with prior evidence suggesting that the impact on fruit consumption is relatively common and increase of vegetable consumption among children harder to achieve (Evans et al., 2012; Savoie-Roskos et al., 2017).

While the finding that snack time consumption increased by 25 grams of fruit and 14 grams of vegetables may seem modest, 25 g is ¼ cup of fruit which equates to one serving for 3–5 year olds according to the Child and Adult Care Food Program (CACFP) guidelines (Food and Nutrition Service and U. S. Department of Agriculture, n.d.). Similarly, 14 g of vegetables is slightly less than1/8 cup, which is equivalent to a half serving of vegetables. If scaled across four daily snacks and meals (morning snack, lunch, afternoon snack, and supper), the total may equate to 4 servings of fruit and 2 servings of vegetables bringing children to recommended daily consumption of three cups of FV daily (U.S. Department of Agriculture, 2021).

The non-significant results regarding FV liking may reflect a common developmental pattern. Research shows that neophobia (the rejection of new tastes) increases gradually between the ages of 2 and 5 years and decreases in subsequent years (Cooke et al., 2003). As children grow, their understanding of what to eat or not eat increases as they are encouraged to taste and make decisions by themselves. With the development of cognitive skills (at about 7 years of age), children are able to make rational choices based on previous experiences (Birch et al., 1987). It may be the case that the gardening intervention does not affect FV liking. Alternatively, the non-significant result regarding FV liking may be explained by limits to construct validity inherent in our measures, described below.

### Study strengths and limitations

#### Strengths

This study makes several contributions. First, it examines hands-on gardening in a vulnerable, under-studied population: children within low-income communities who attend childcare. This population is not only at risk for poor diet (Lorson et al., 2009) and overweight (Robert Wood Johnson Foundation, 2021) but is seldom the focus of research. Moreover, while early interventions have the potential to affect change over the lifecourse (Wethington, 2005; García et al., 2020), environment and behavior studies of preschool children remain a critical gap in the literature.

The internal validity of this study is bolstered by its RCT design, which allows us to rule out multiple alternative explanations or threats to internal validity. An additional strength is construct validity. Established, age-appropriate measures were employed for all dependent variables. FV consumption, which is particularly challenging to measure in young children (Rockett and Colditz, 1997; Warren et al., 2003; Livingstone et al., 2004; Magarey et al., 2011), is objectively measured via pre- and post-snack time weighing of FV, avoiding the quagmire of threats to construct validity associated with self-report dietary data (Livingstone and Robson, 2000).

#### Limitations

This study is not without limitation. Regarding external validity, it is possible that study findings may not be readily generalizable to other climate or geographical areas. While overall, the construct validity of this study is strong – with the use of valid, reliable, age-appropriate measures – there are still inevitable limitations. The measurement of FV consumption during tasting sessions (snack times) means that the measure may not correspond directly to daily dietary intake of FV. The measure of FV liking, which employed visual images of fruit and vegetables presented on a touch-screen tablet (1 day before tasting vegetables and 2 days before tasting fruit), may have relatively weak construct validity, particularly for such young children who may not have the cognitive ability to remember whether they have eaten the item before and whether they did indeed like it. Thus, the FV liking measure may have been more effective if it had followed the tasting sessions or was synchronous with tasting. In this way, children might have been more likely to report whether they liked to eat the FV (rather than, perhaps, merely whether they ‘liked’ the visual image). The construct validity limitations regarding the FV liking may in fact underlie the non-significant effects on FV liking. In other words, the compromised construct validity may, in turn, have affected the statistical (and internal) validity of this facet of the study – making a Type 2 (“miss”) more likely with respect to the effects of the intervention on children’s liking of FV.

This study did not examine the possible mediating mechanisms that would illuminate the explanatory pathways by which the gardening intervention affects FV outcomes. Similarly, the examination of moderators (or “effect modifiers”) was beyond the scope of this study.

Additional limitations are presented by the inherent challenges of working with childcare centers serving low-income families whose working schedules are tightly connected to their services (Sandstrom and Chaudry, 2012; VanLeer et al., 2021). The unstable nature of low-income jobs often has an impact on children’s childcare attendance due to relocation, changes in parent schedules, lack of transportation, or other issues. Like most childcare centers serving low-income communities, the centers that participated in this study tended to be understaffed and have high turnover of teachers and leadership (Grunewald et al., 2022). Due to these factors, this study experienced attrition of participating children (Cosco et al., 2021).

### Implications

The childcare gardening intervention increased children’s dietary intake, modestly but significantly, raising the question: does hands-on gardening infrastructure, and related pedagogical programming, deliver a viable return on investment? The approximate installation cost of each COLEAFS garden was $1,500 for materials and labor (2018 dollars) – a small investment compared to the renovation cost ($50 K – $100 K) of a complete outdoor learning environment using best practices (Moore and Cosco, 2021). Garden-based learning offers a rewarding opportunity for classroom teachers to directly engage children in an adaptable interdisciplinary outdoor pedagogy (STEAM: science, technology, engineering, art, mathematics (Vandermaas-Peeler and McClain, 2015; Linder and Eckhoff, 2020).

An additional upfront cost for training may also be needed to help teachers learn about gardening basics (i.e., choosing fertile soil and appropriate seeds, identifying adequate orientation with sufficient sunlight, preparing containers, and following irrigation schedules). A starter garden can be as modest as tomato and basil plants for a simple salad. The power of experiential garden-based learning during the preoperational preschool years (Zeinstra et al., 2007), is underscored by Piaget’s seminal insistence that for children to understand something they “must do their own experimenting, their own research” (Piaget, 1972, p. 27). Skill acquisition (García et al., 2018) and cognitive development (Zeinstra et al., 2007), may support dietary impacts that scale up as a lifecourse health benefit (Wethington, 2005).

Garden interventions in disadvantaged communities may provide an opportunity to reduce disparities in healthy eating, particularly for African American (Sharma et al., 2014) and Latino children (Davis et al., 2011). Knowledge of gardening acquired by young, disadvantaged children attending childcare (Zeinstra et al., 2007) may also help to level the “healthy playing field” to enable the equigenic effect of contact with nature (Mitchell, 2013; Jordan, 2020; Wells, 2021). Because the COLEAFS context was low-resource communities with high percentages of subsidized families and racial minorities, the impacts may be amplified compared to similar interventions in advantaged communities (Elango et al., 2016).

Since U.S. childcare systems are highly regulated and policy sensitive, state-level policy changes can rapidly ripple across systems. If early childhood gardening is considered a potentially influential healthy eating strategy, informing state leadership with change-provoking evidence may be an effective strategy. Policy pathways have already been laid by innovative, US state-level assessment models emphasizing experiential learning and gardening. Included are the NC Foundations for Early Learning (North Carolina Foundations Task Force, 2013), South Carolina Early Learning Standards (South Carolina Early Learning Standards Interagency Stakeholder Group, 2017), and the Texas Prekindergarten Guidelines (University of Texas System and Texas Education Agency, 2015).

Findings from this study add evidence that may support licensing regulations, assessment protocols, accreditation standards, and community college courseware to adopt garden-based learning as a convincing driver for early childhood healthy nutrition. Adoption may scale up childcare systems as an effective health and wellness intervention that considers investment in gardening as a focal target for social return on investment (SROI) (Hamelmann et al., 2017).

### Future research

Opportunities for future research are many and varied. With sufficient resources, a longer longitudinal study might follow children after their time in childcare, into elementary school and beyond, to gain a more complete understanding of influence of early gardening experiences on dietary trajectories. Similarly, studies might further embrace the bioecological model (Bronfenbrenner, 1979), to examine the influence of key microsystems and how these contexts interact to affect a child’s dietary intake (Story et al., 2008). Prior research suggests that a school garden intervention may have effects that carry over to the home environment (Wells et al., 2018) but there is a need for a broader understanding of the interplay among settings.

Future studies might focus more explicitly on mediating mechanisms to illuminate the explanatory pathways from intervention to dietary intake. A focus on mediation not only enriches a conceptual understanding of the processes contributing to dietary habits, but also provides practical leverage points, expanding the range of targets for intervention. Possible mediating mechanisms linking a garden intervention to FV consumption include exposure to FV (Cooke, 2007) and the availability (i.e., presence) of FV (Jago et al., 2007). It is plausible that some mediators stretch beyond the childcare center to other contexts of the child’s life (Bronfenbrenner, 1979; Wells et al., 2018). For example, parents’ awareness of, or involvement in a childcare-based gardening intervention may lead them to become curious about FV or motivated to improve diet at home. Similarly, children, following their exposure to FV via gardening, might increase their “asking skills” related to FV, when eating or shopping with parents (Askelson et al., 2019). Thus, parents and the home environment may be among the possible spokes by which a childcare garden could affect change.

Relationships between FV availability and FV home gardening with children may be a potent research direction. The complex, many-layered process of home food management modeled by Campbell and Desjardins (1989), stresses assessment of the family context as essential for improving nutritional health of low-income families and their children and underscores proximal availability of food as a potentially strong mediator. Gardening at home is a traditional activity of family contexts around the world and has, for example, been associated with Filipino preschool-aged child diet diversity and frequency of vegetable consumption (Cabalda et al., 2011). Hands-on gardening for children at home (even as modest as veggies and herbs in containers) may strengthen proximal availability and provide significant experiential learning, especially if linked to gardening experiences at preschool. Center-home FV synergy may enhance children’s familiarity with FV, increase home experience and FV availability, impact positive home consumption, and expand informed FV conversations at the grocery store (Baranowski et al., 2000).

Data for the study reported here were gathered in the Piedmont region of North Carolina, where the warm and cool growing seasons extend through most of the year. Replication in different climatic zones would provide a necessary test of external validity but also may offer valuable information regarding the practicalities of preschool FV gardening under more extreme climatic conditions, including, for example, glazed indoor spaces to extend the growing season in northern latitudes.

Taking advantage of new technologies, big data analyses (crossing pediatric health and demographics data with environmental opportunities for gardening), might offer a pathway to maximize use of existing garden installations or identify locations to create programs in disadvantage communities (Altaweel, 2022; ArcGIS, 2022).

## Data availability statement

Full datasets are not readily available because subjects were young children and parent consents did not include permission to share information with researchers beyond the study team. Further inquiries can be directed to the corresponding author.

## Ethics statement

The studies involving human participants were reviewed and approved by North Carolina State University Institutional Review Board (IRB) according to the Declaration of Helsinki guidelines on research ethics. Protocol approval #5908. Written informed consent to participate in this study was provided by the participants’ legal guardian/next of kin.

## Author contributions

NGC, NMW, LSG, MM, and RCM contributed to research design and measurement selection. NGC and NMW led the manuscript preparation and writing. DZ and TX led the data analysis. RCM contributed knowledge on garden design and sustainable practices. NCG oversaw data collection and intervention implementation. MM contributed to the study preparation and Year 1 data collection. All authors contributed to the manuscript and reviewed the submitted version.

## Funding

The study was supported by the United States Department of Agriculture (USDA) National Institute of Food and Agriculture (NIFA), Agriculture and Food Research Initiative (AFRI) Competitive Grant No. 2017-68001-26354.

## Conflict of interest

The authors declare that the research was conducted in the absence of any commercial or financial relationships that could be construed as a potential conflict of interest.

## Publisher’s note

All claims expressed in this article are solely those of the authors and do not necessarily represent those of their affiliated organizations, or those of the publisher, the editors and the reviewers. Any product that may be evaluated in this article, or claim that may be made by its manufacturer, is not guaranteed or endorsed by the publisher.
