# Supplementary figures and images for: Hands-on childcare garden intervention: A randomized controlled trial to assess effects on fruit and vegetable identification, liking, and consumption among children aged 3–5 years in North Carolina
Source: Front Psychol. 2022 Nov 10;13:993637. doi: 10.3389/fpsyg.2022.993637 (PMC9685661; doi:10.3389/fpsyg.2022.993637)

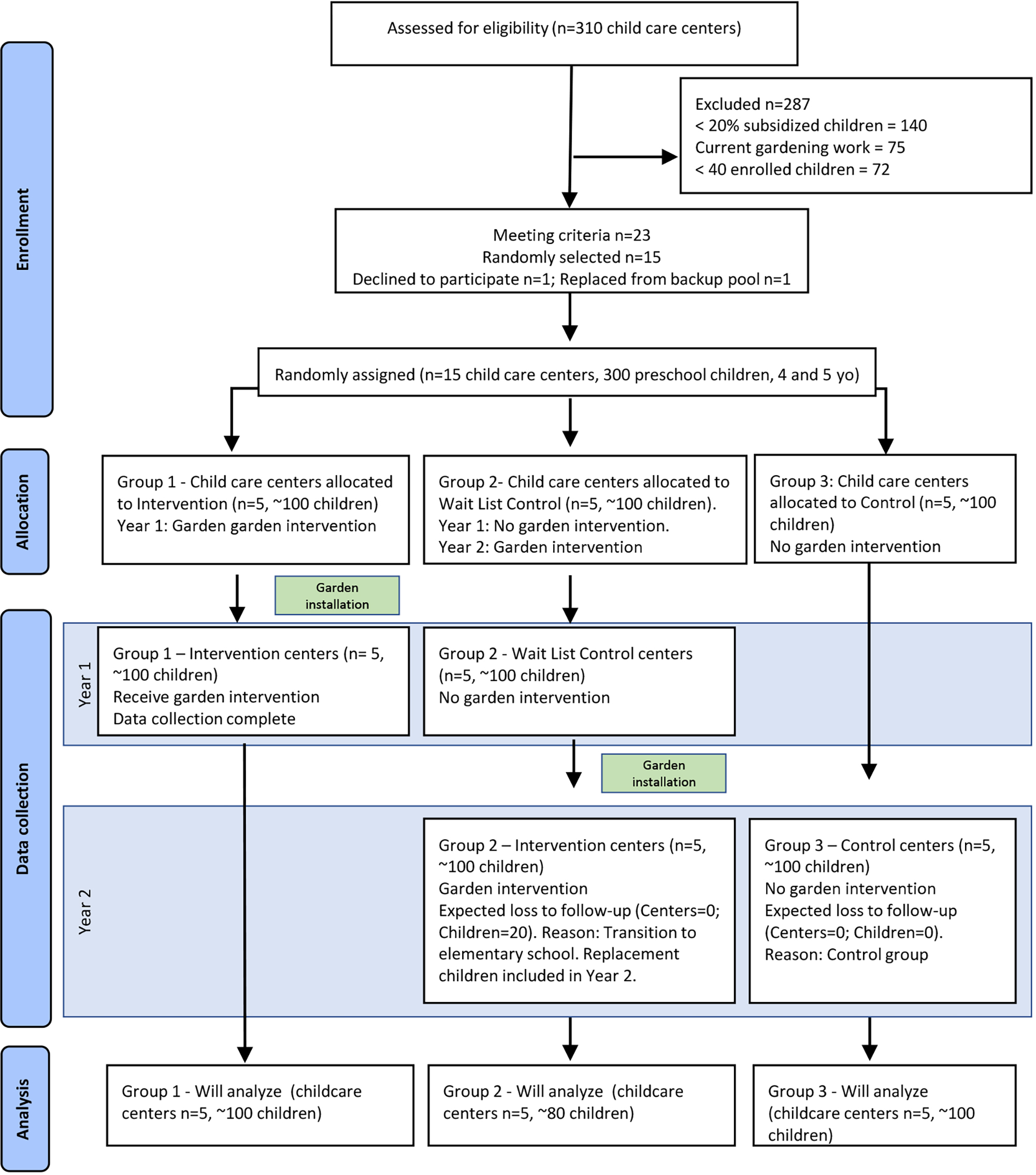

Supplement: Supplementary file 1 [file Image_1.JPEG]
